# Supplementary figures and images for: Laser-Induced Ion Formation and Electron Emission from a Nanostructured Gold Surface at Laser Fluence below the Threshold for Plasma Formation
Source: Nanomaterials (Basel). 2023 Feb 2;13(3):600. doi: 10.3390/nano13030600 (PMC9919040; doi:10.3390/nano13030600)

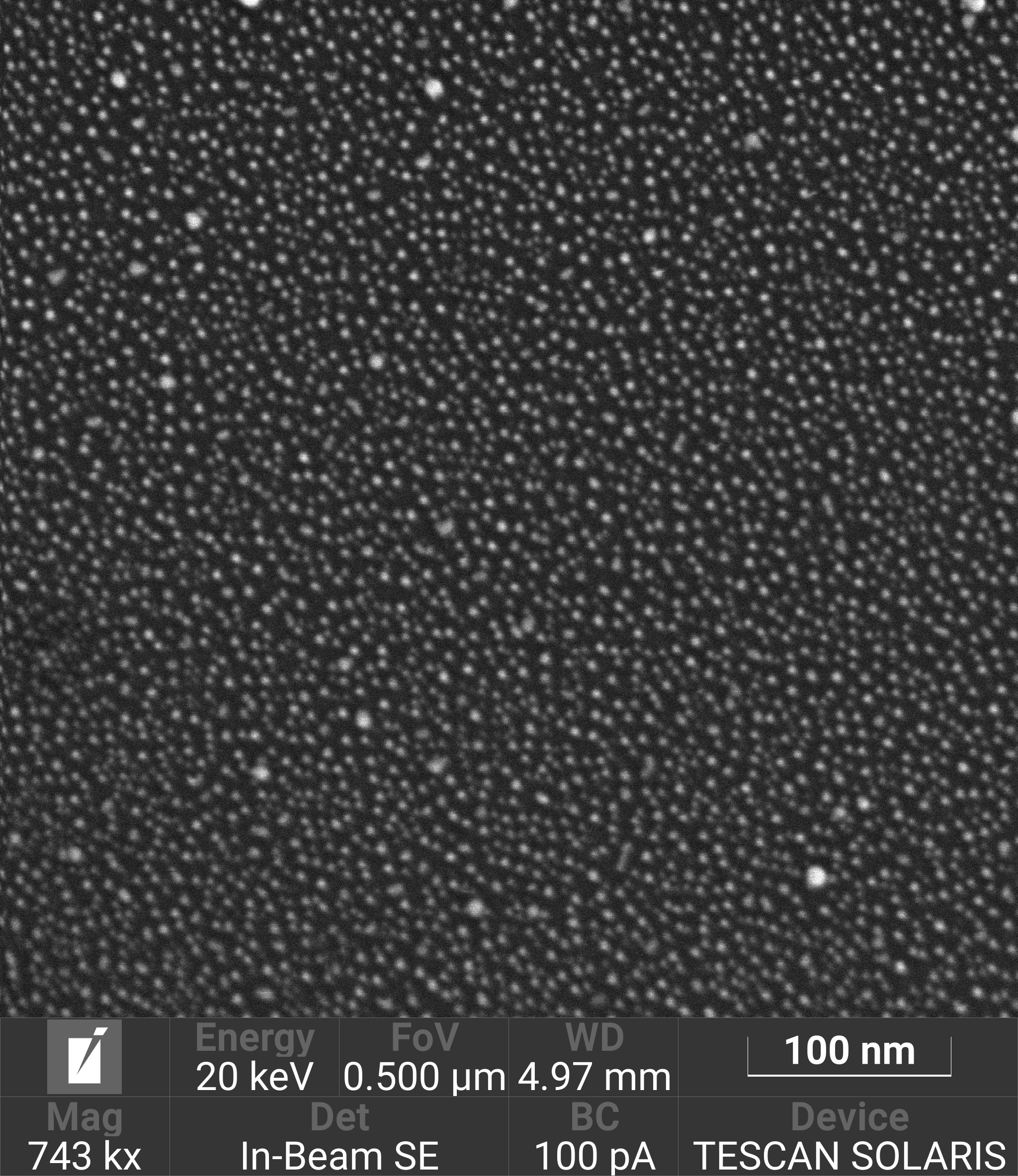

Supplement: Supplementary file 1 [file nanomaterials-13-00600-s001.zip › figure S1.tif]

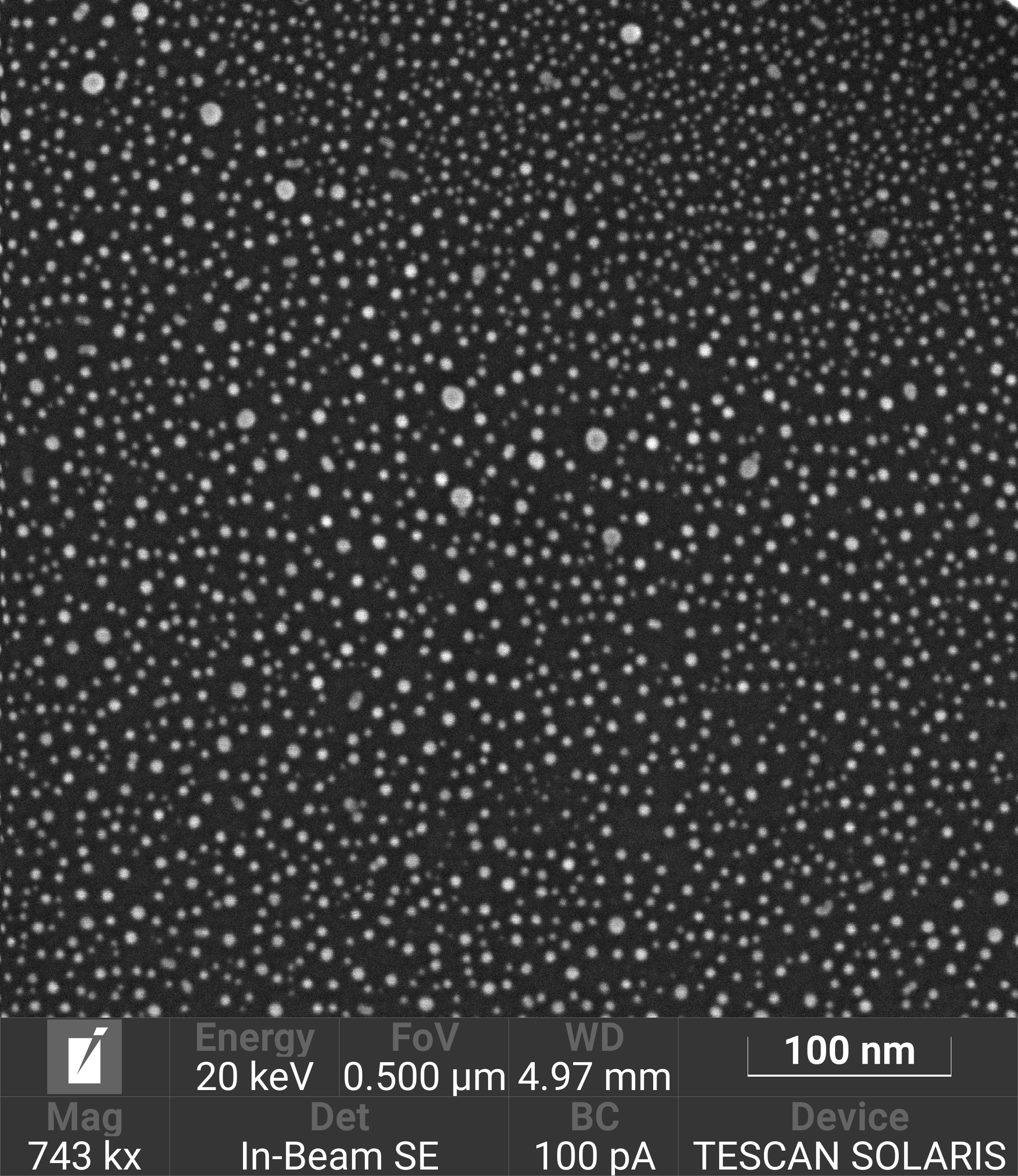

Supplement: Supplementary file 1 [file nanomaterials-13-00600-s001.zip › figure S2.tif]
